# Mediastinal tuberculosis presenting as traction diverticulum of the esophagus

**DOI:** 10.4103/1817-1737.33703

**Published:** 2007

**Authors:** Anurag Rastogi, Dinesh Sarda, Paras Kothari, Bharati Kulkarni

**Affiliations:** *Department of Paediatric Surgery, L.T.M.M.C. and Gen. Hospital, Mumbai, India*

**Keywords:** Mediastinal tuberculous lymphadenopathy traction diverticulum

## Abstract

A 7-year-old male presented with history of low-grade fever, epigastric pain and dysphagia. Ultrasound of abdomen and thorax revealed presence of paraesophageal lymphadenopathy. ‘Barium swallow’ and computerized tomography scan thorax with oral contrast suggested a provisional diagnosis of paraesophageal diverticulum. Esophagoscopy was normal. Endoscopic ultrasonography with biopsy confirmed tuberculosis. The patient was started on four-drug antitubercular treatment.

Mediastinal tuberculous lymphadenopathy is common, but esophageal tuberculosis (TB) is rare, even in endemic regions. It can be primary or secondary due to rupture of caseating paraesophageal lymph nodes. Patients usually present with epigastric pain and dysphasia, along with constitutional symptoms of TB. A high index of clinical suspicion supported by investigations like computerized tomography (CT) scan chest, esophagoscopy and endoscopic ultrasound help in diagnosis. Most cases respond successfully to antituberculous therapy. Few may require surgical management.

## Case Report

A 7-year-old male presented with history of acute epigastric pain and nonbilious vomiting of 1-day duration. There was history of low-grade fever in the evening for 1 month. After 1 week of admission, he developed dysphagia for solids at the lower thoracic level. Retrospective analysis did not confirm family history or history of contact with tuberculosis. He was immunized till age.

**Figure 1:**
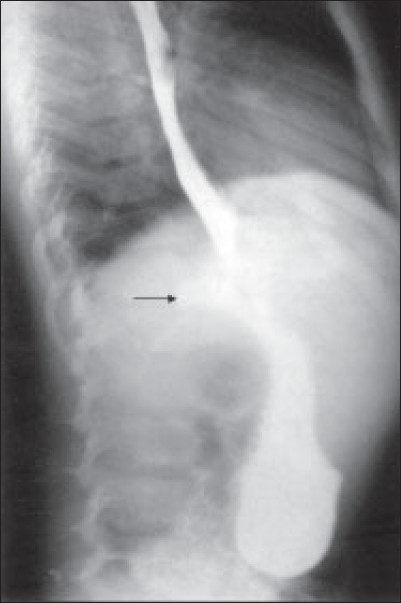
‘Barium swallow’ showing a blind-ending tract near the lower third of esophagus (Arrow)

**Figure 2:**
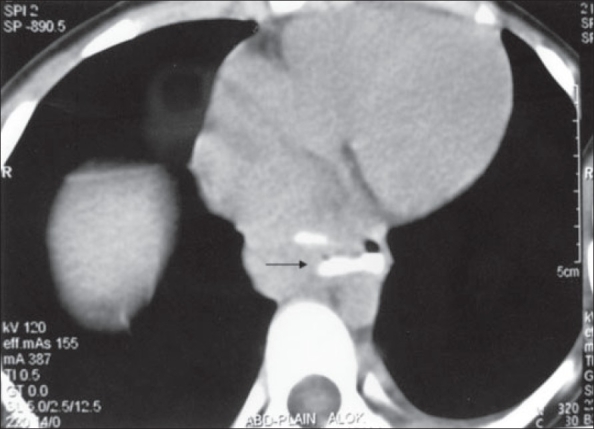
Contrast-enhanced CT scan showing significant luminal compromise and a diverticulum was seen arising from the lower esophagus (Arrow)

**Figure 3:**
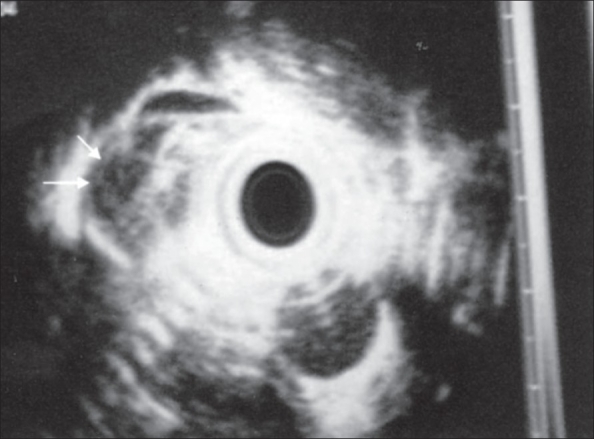
Endoscopic ultrasound showing a large sub-carinal lymph node with central necrosis and submucosal thickening with a large paraesophageal lymph node adherent to the esophagus near gastroesophageal junction

On examination, vitals were stable. Chest examination was normal. Per abdominal examination revealed epigastric tenderness with a vague mass. Hemogram was normal. ESR was 22 mm at the end of the first hour. Mantoux test was negative. Chest X ray revealed a faint radio-opaque shadow in the lower paraesophageal region. Ultrasonography (USG) abdomen showed a 3 × 3 cm isoechoic lesion in epigastrium, causing mass effect on the posterior surface of esophagus. It was suggestive of enlarged lymph nodes. ‘Barium swallow’ showed a blind ending tract near the lower third of esophagus [Figure 1]. CT scan of the mediastinum and upper abdomen with oral contrast revealed a well-defined hypodense lesion with central necrosis in lower paraesophageal region. Esophageal wall was edematous with significant luminal compromise, and a diverticulum was seen arising from the lower esophagus [Figure 2]. On esophagoscopy, the mucosa was normal. No stricture or mouth of the sinus tract could be identified. A probable diagnosis of tuberculosis was entertained. Endoscopic ultrasound showed a) 3-cm size sub-carinal lymph node with central necrosis and b) submucosal thickening with a large paraesophageal lymph node adherent to the esophagus near the gastroesophageal junction [Figure 3]. Fine needle aspiration cytology from the nodal mass confirmed diagnosis of TB, and the patient was started on four-drug antituberculous treatment.

Follow-up CT scan with contrast done after 2 months showed significant decrease in size of the hypoechoic lesion. Antitubercular treatment was given for 6 months. The patient is asymptomatic on follow-up at 1 year.

## Discussion

TB of the esophagus may be primary or secondary. Mechanisms of secondary tubercular involvement of the esophagus are (1) swallowed sputum in patients having advanced open TB; (2) direct involvement from tuberculosis involving the lungs, mediastinal lymph nodes or thoracic spine; (3) retrograde lymphatic spread and (4) blood borne.[1] Three macroscopic types are recognized: hypertrophic, granular and ulcerative.[1] Common presenting symptoms are pain and dysphagia. Our patient presented with acute symptoms of pain and vomiting and later developed dysphagia. Rupture of mediastinal TB lymph node into esophagus is rare.[2] Mid-esophagus is the commonest site of involvement, near bifurcation of trachea, due to close proximity to mediastinal lymph nodes.

Diagnosis is suspected after investigations like ‘barium swallow,’ CT thorax[3] and confirmed by endoscopic biopsy.[4,5] Radiological abnormalities seen in ‘barium swallow’ are extrinsic compression, traction diverticula, strictures, sinus/ fistulous tracts, kinking and pseudo-tumor mass of esophagus – in decreasing order of frequency.[1] Diminished motility and mucosal irregularity may also be seen. CT chest gives most complete delineation of the tuberculous mediastinal lymphadenopathy and the fistulous tract extending from the esophagus into the nodal mass. The clinical suspicion in our case arose on correlating dysphagia with USG and CECT scan findings of lymph node enlargement. ‘Barium swallow’ and CECT scan were able to pick up the fistulous tract. On esophagoscopy, ulcer with undermined edges is a common finding, and esophageal sinus or fistulous opening may be seen.[6] This finding was elusive in our case. Diagnosis was clinched by endoscopic ultrasound-guided aspiration of the necrotic lymph nodes. Majority of the patients respond well to antitubercular treatment. Our patient was stable and hence he was started on four-drug antitubercular treatment, to which he responded very well. Few may require surgery, but as the last option.[7] The surgery consists of paraesophageal drainage of the mediastinal abscess and esophageal diversion.[6]

The above case has been reported because of its rarity. We would like to highlight that tuberculosis still remains a part of differential diagnosis of lymphadenopathy in developing countries.
